# Specific Synthesis of Neurostatin and Gangliosides *O*-Acetylated in the Outer Sialic Acids Using a Sialate Transferase

**DOI:** 10.1371/journal.pone.0049983

**Published:** 2012-12-03

**Authors:** Lorenzo Romero-Ramírez, Isabel García-Álvarez, Ramón Campos-Olivas, Michel Gilbert, Marie-France Goneau, Alfonso Fernández-Mayoralas, Manuel Nieto-Sampedro

**Affiliations:** 1 Unidad de Neurología Experimental, Hospital Nacional de Parapléjicos, Toledo, Spain; 2 Instituto Cajal de Neurobiología, CSIC, Madrid, Spain; 3 Instituto de Química Orgánica General, Madrid, Spain; 4 Spectroscopy and NMR Unit. Structural Biology and Biocomputing Programme, Spanish National Cancer Center (CNIO), Madrid, Spain; 5 Human Health Therapeutics, National Research Council Canada, Ottawa, Ontario Canada; Faculdade de Medicina, Universidade de São Paulo, Brazil

## Abstract

Gangliosides are sialic acid containing glycosphingolipids, commonly found on the outer leaflet of the plasma membrane. *O*-acetylation of sialic acid hydroxyl groups is one of the most common modifications in gangliosides. Studies on the biological activity of *O*-acetylated gangliosides have been limited by their scarcity in nature. This comparatively small change in ganglioside structure causes major changes in their physiological properties. When the ganglioside GD1b was *O*-acetylated in the outer sialic acid, it became the potent inhibitor of astroblast and astrocytoma proliferation called Neurostatin. Although various chemical and enzymatic methods to *O*-acetylate commercial gangliosides have been described, *O*-acetylation was nonspecific and produced many side-products that reduced the yield. An enzyme with *O*-acetyltransferase activity (SOAT) has been previously cloned from the bacteria *Campylobacter jejuni*. This enzyme catalyzed the acetylation of oligosaccharide-bound sialic acid, with high specificity for terminal alpha-2,8-linked residues. Using this enzyme and commercial gangliosides as starting material, we have specifically *O*-acetylated the gangliosides’ outer sialic acids, to produce the corresponding gangliosides specifically *O*-acetylated in the sialic acid bound in alpha-2,3 and alpha-2,8 residues. We demonstrate here that *O*-acetylation occurred specifically in the C-9 position of the sialic acid. In summary, we present a new method of specific *O*-acetylation of ganglioside sialic acids that permits the large scale preparation of these modified glycosphingolipids, facilitating both, the study of their mechanism of antitumoral action and their use as therapeutic drugs for treating glioblastoma multiform (GBM) patients.

## Introduction

Gangliosides are sialic acid containing glycosphingolipids, commonly found on the outer leaflet of plasma membranes. As structural components of neural cell membranes, gangliosides play important roles in cell adhesion [1], cell recognition [2] signal transduction [3] and neural development [4]. Many *O*-acetylated gangliosides have been described in mammalian and non-mammalian tissues [5]. *O*-acetylation of hydroxyl groups is one of the most common modifications of sialic acids and exist in nature at the C-4, 7, 8 and 9 hydroxyl groups [6], [7], [8]. This comparatively small change in the ganglioside structure causes major changes in their physiological properties, resistance to sialidase hydrolysis and lectin binding [9]. When the ganglioside GD1b was *O*-acetylated in the outer sialic acid, it became the potent inhibitor of astroblast and astrocytoma proliferation called Neurostatin [10], [11].

We [12], [13] and others [14], [15] have developed several synthetic methods for the enzymatic and chemical *O*-acetylation of commercial gangliosides, although the yields obtained were poor and the reactions led to many similar products that were very difficult to purify.

In mammals, two enzyme systems are known to be responsible for *O*-acetylations: AcCoA:sialate-4-*O*-acetyltransferase (EC 2.3.1.44) [16] and AcCoA:sialate-7(9)-*O*-acetyltransferase (EC 2.3.1.45) [17], [18]. Identification of the sialate-*O*-acetyltransferase (SOAT) gene has been possible in some microorganisms [19], [20], [21], but not yet in eukaryotic cells [22].

A SOAT transferring acetyl groups exclusively to C-9 in the fluorescent ganglioside analog GD3-FCHASE was isolated and cloned from *Campylobacter jejuni* [23]. Using this enzyme, we present here a new method for the specific *O*-acetylation of outer sialic acids in gangliosides at the C-9 hydroxyl group, permitting the large scale preparation of these modified glycosphingolipids.

**Figure 1:**
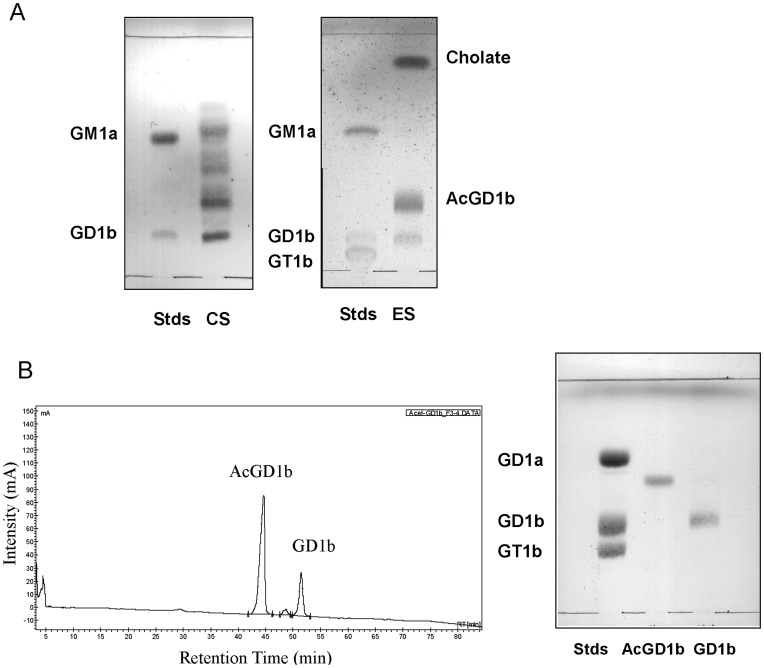
SOAT catalyzes the synthesis of AcGD1b. (**A**) TLCs comparing the products obtained using two methods of *O*-acetylation for GD1b: chemical synthesis (CS) with trimethylortoacetate and enzymatic synthesis (ES) with SOAT. (**B**) HPLC chromatogram of the enzymatic reaction and TLC of products purified by HPLC.

## Materials and Methods

### Purification of Recombinant SOAT

*Escherichia coli* AD202 containing the construct CJL-130 (Orf11) from *Campylobacter jejuni* ATCC 43446 in pCWori+ [23], was grown in 2 YT medium containing 150 µg/ml ampicillin and 2 g/liter glucose. The culture was incubated at 37°C until A_600_ = 0.35, induced with 1 mM isopropyl 1-thio-β-D-galactopyranoside, and incubated overnight at 20°C. The cells were resuspended in buffer A containing 20 mM Tris pH 7.5, 200 mM NaCl, 5 mM β-mercaptoethanol, 1 mM EDTA and a protease inhibitor cocktail (Sigma #P2714). The cells were broken using an Avestin C5 Emulsiflex cell disruptor (Avestin, Ottawa, Canada) and the extract was clarified by centrifugation at 27,000×*g* for 30 min. The MalE-Orf11 fusion protein (SOAT) was purified by affinity chromatography on amylose resin, following the manufacturer’s instructions (New England Biolabs, Beverly, USA). The clarified extract was loaded on a column containing 25 ml of amylose resin at a flow rate of 1 ml/min. The column was washed with 35 ml of buffer A and then developped with 30 ml of buffer B (buffer A with 10 mM maltose and 10% glycerol). The 1.5 ml fractions were analysed by SDS-PAGE on a 12% gel. The fractions containing the SOAT were pooled and dialysed o/n with a buffer containing 10 mM Hepes pH 6.5 and 20 mM NaCl. The dialysed SOAT was lyophilized in aliquots and stored at −20°C until use. SOAT activity was measured in each culture batch by measuring the production of 9-*O*-acetyl-GD3-FCHASE product using GD3-FCHASE as acceptor [23]. One unit of SOAT activity was defined as the amount of enzyme that produces 1 µmol of 9-*O*-acetylated GD3-FCHASE in 1 min.

**Figure 2:**
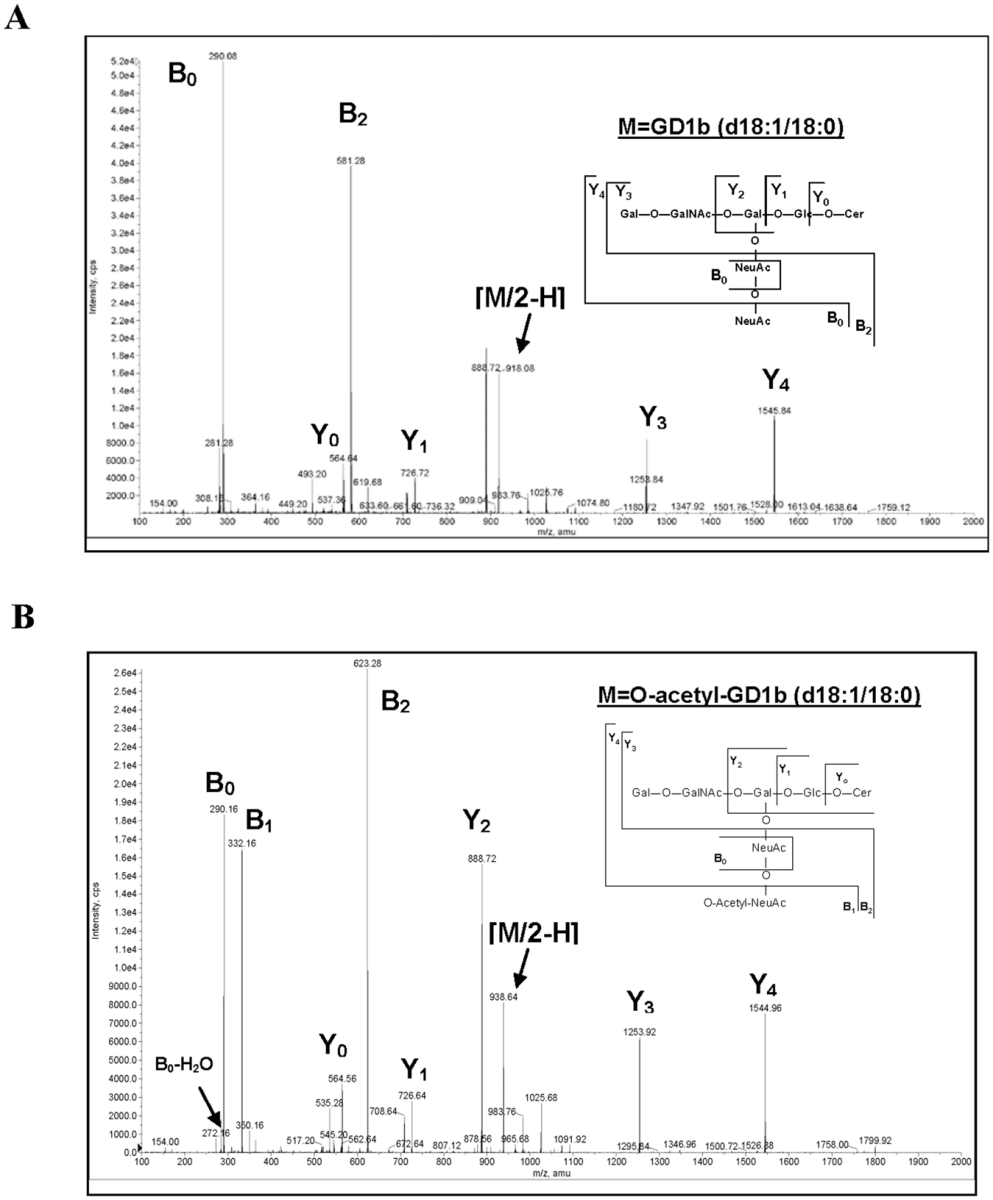
SOAT *O*-acetylates GD1b in the alpha-2,8 bound sialic acid. Negative electrospray-MS spectra of GD1b (**A**) and AcGD1b (**B**) gangliosides species that contain a ceramide with a LCB of 18∶1 and F.A. of 18∶0. (d18∶1/18∶0). The m/z values are indicated for every peak.

### Synthesis with SOAT of *O*-acetylated Gangliosides

Different commercially available gangliosides (GD1a, GD1b, GM1a, GM2 or GT1b from Santa Cruz Biotechnology, Santa Cruz, USA) were *O*-acetylated with the help of the *O*-acetyltransferase (SOAT) isolated from *C. jejuni.* The enzymatic reaction was modified from that described previously [23]. Briefly, the ganglioside (1 mM) was dissolved in MES buffer (50 mM) pH 6.5 or pH 7, containing acetyl-Coenzyme A (1 mM), MgCl_2_ (10 mM) and dithiothreitol (1 mM) (Fluka, Buchs, Germany). Various concentrations of sodium cholate (Sigma-Aldrich, Steinheim, Germany), ranging from 0 to 0.2% (w/v) were added to the reaction. SOAT (100 mU) was added and the reaction mixture was incubated at 37°C for 3 hours with stirring (300 rpm) and stopped by adding methanol (50% final). The enzymatic reaction was desalted by preparative reverse-phase chromatography on a C18 Sep-Pak cartridge (Waters**,** Millford, USA) [24], dried on a speed-vac evaporator and stored at −20°C. Reaction products were monitored by TLC (Thin Layer Chromatography) on aluminum plates coated with silica gel 60 (Merck, Darmstadt, Germany). Samples were dissolved in methanol and applied by hand to TLC plates or using a Linomat 5 system (Camag, Muttenz, Switzerland). TLCs were developed with Chloroform/Methanol/0.2% CaCl_2_ (45/55/10, v/v/v), using as control a standard ganglioside mixture (GT1b, GD1b, GD1a and GM1, Santa Cruz Biotechnology) or monosialoganglioside mix (GM1a, GM2 and GM3, Matreya Inc, Pleasant Gap, USA). The standard gangliosides lane and the reaction mixture lane were visualized by spraying with 3.5% sulphuric acid in methanol, followed by heating at 120°C.

**Figure 3:**
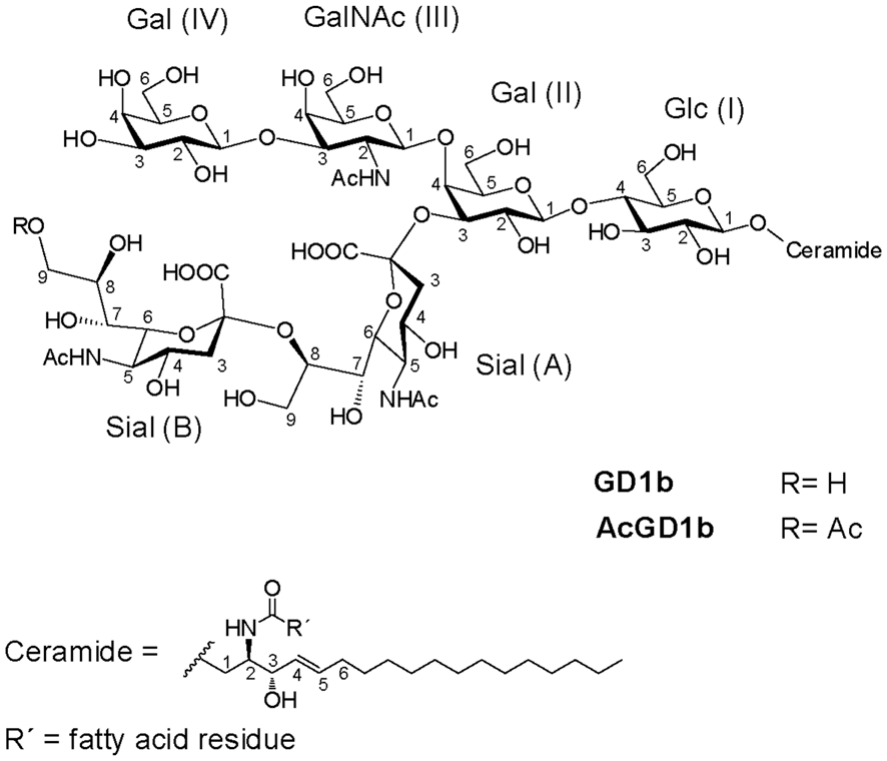
Chemical structures of GD1b and AcGD1b (Neurostatin).

### Purification of Compounds

The desalted solutions obtained from the reactions were purified by silica gel column chromatography or preparative TLC. The samples were applied to a silica gel column (1.4 g, Merck 60, 0.040–0.063 mm) and eluted with a gradient of MeOH-CHCl_3_-H_2_O from (55∶45:0, v/v/v) to (55∶45:10). The fractions obtained were concentrated on a speed-vac evaporator and monitored by TLC as described above.

**Figure 4:**
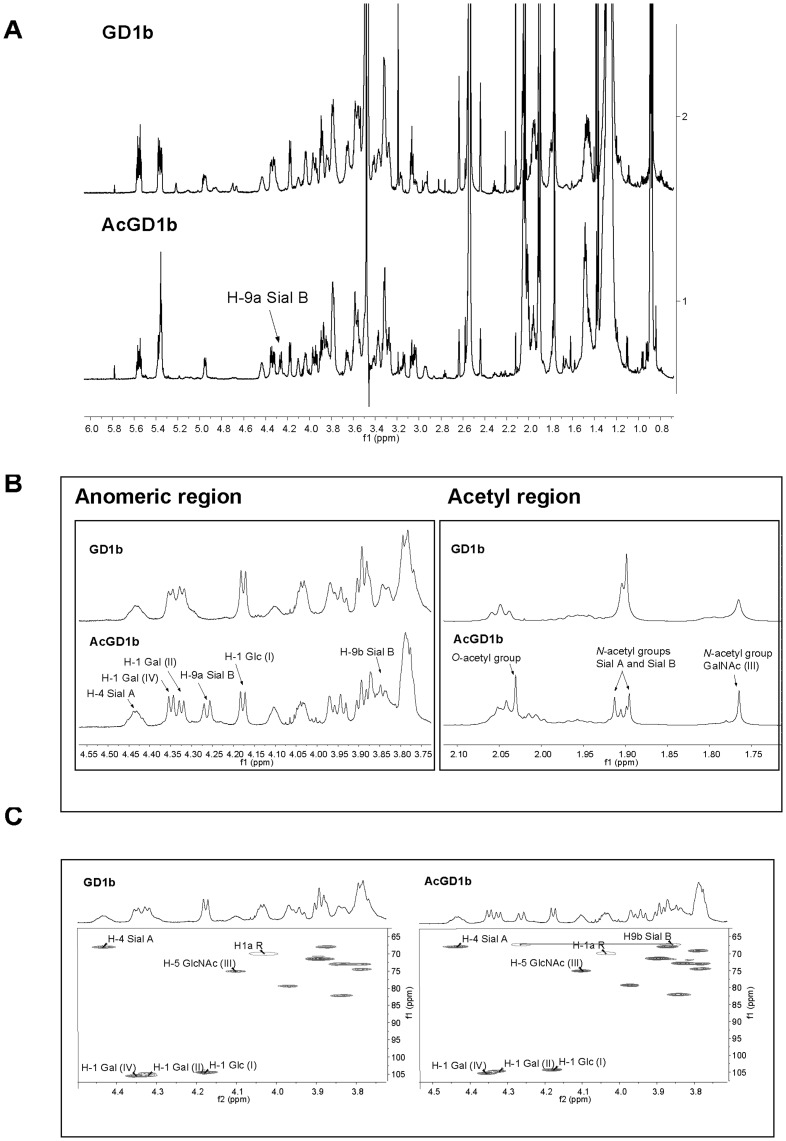
SOAT *O*-acetylates GD1b in the C-9 position of the alpha-2,8 bound sialic acid. (**A**) Comparison of the 700 MHz ^1^H-NMR spectra of **GD1b** and **AcGD1b** in DMSO-d_6_/D_2_O 98∶2 at 298 and 295 K, respectively. (B) Magnified ^1^H-NMR spectra in the anomeric and acetyl region of **GD1b** and **AcGD1b**. (C) Comparison of the anomeric region in the 2D ^1^H-^13^C multiplicity-edited HSQC spectra of **GD1b** and **AcGD1b**. In this spectrum signals arising from CH_3_ and CH groups have different sign (positive) to those of CH_2_ groups (negative). Cross peaks are positive (several contours) for CH_2_ groups and negative (one contour) for CH and CH_3_ groups. In both cases the corresponding 1D ^1^H specrum is shown above the 2D spectrum.

For TLC purification, the desalted solution obtained from the reaction was applied to the TLC as described above. Three lanes of the TLC containing standard gangliosides, a lane with a small amount of the reaction mixture and an additional lane with the reaction mixture for purification were run in parallel to determine the R_f_ of the *O*-acetyl compounds. The standard gangliosides lane and the lane with a small amount of the reaction mixture were visualized by spraying with 3.5% sulphuric acid in methanol, followed by heating at 120°C. After identification of the compounds by their R_f_ value, the desired bands from the reaction mixture for purification were scraped and eluted with methanol (2 h at 4°C, with agitation). The silica gel was removed by centrifugation (12,000×*g*, 5 min at 4°C) and by a preparative reverse-phase chromatography on a C18 Sep-Pak cartridge (Waters**,** Millford, USA) as described [25], concentrated on a speed-vac evaporator and stored at −20°C.

**Figure 5:**
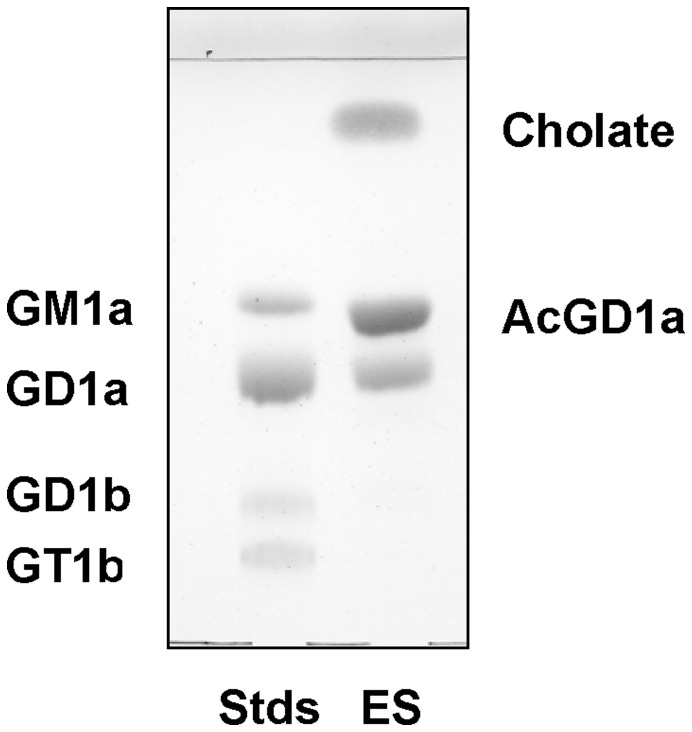
SOAT catalyzes the synthesis of AcGD1a. TLC of the products obtained in the reactionof GD1a with SOAT (ES).

HPLC was used to determine the yield of the reaction and the purity of the samples. All analyses were performed using a Jasco Pu-2089 Plus, Dual Gradient Pump chromatograph, equipped with an ultraviolet Jasco UV-2075 Plus detector and a 20 µL Rheodyne injector. Data acquisition and processing were accomplished with the Jasco ChromPass Chromatography Data System 1.8.6.1 software. A Kromasil-NH_2_ column (4.6×250 mm, 5 µm particle size) was used as stationary phase. Compounds separation was carried out with a mobile phase made with the following gradient mixture: solvent A, acetonitrile-5 mM sodium phosphate buffer, pH 5.6 (83∶17); solvent B, acetonitrile-20 mM sodium phosphate buffer, pH 5.6 (50∶50) as described [26]. The flow rate was 1.0 ml. min^−1^ and the elution profile was monitored by recording the UV absorbance at 215 nm. Peak assignment of non *O*-acetylated gangliosides was achieved by comparison with commercial standards.

**Figure 6:**
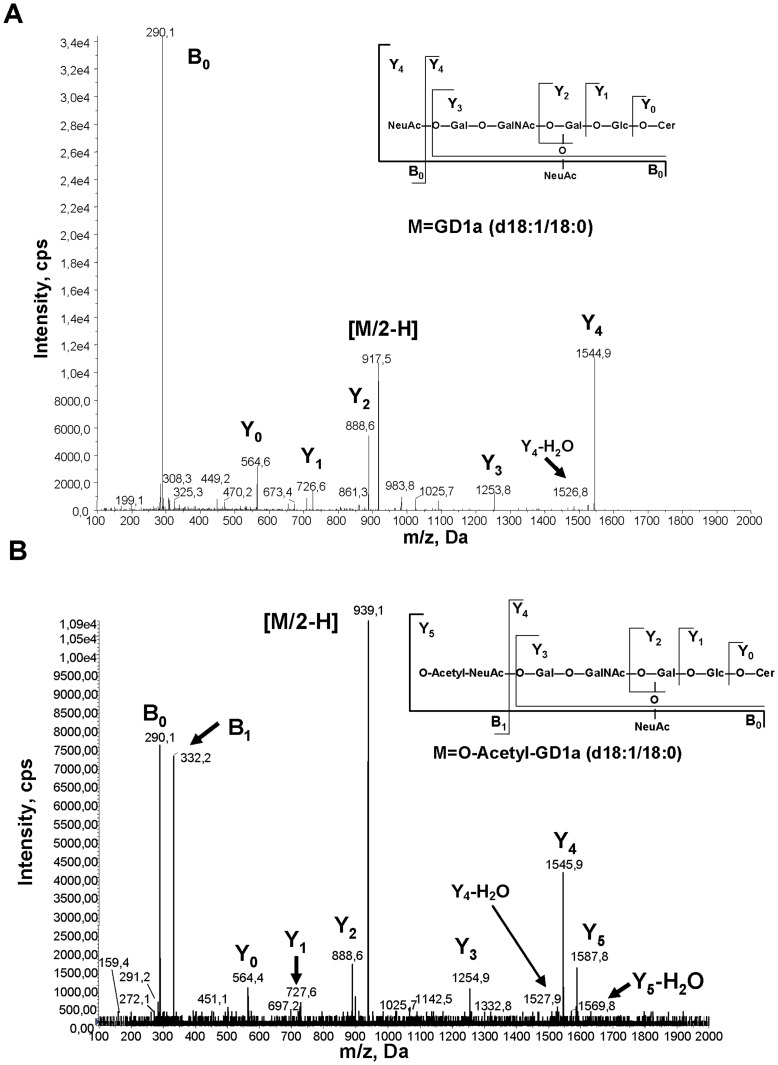
SOAT *O*-acetylates GD1a in a alpha-2,3 bound sialic acid. Negative electrospray-MS spectra of GD1a (**A**) and AcGD1a (**B**) gangliosides species that contain a ceramide with a LCB of 18:1 and F.A. of 18:0. (d18:1/18:0). The m/z values are indicated for every peak.

### Molecular Mass Determination of the Compounds by MALDI-TOF-MS

Samples were dissolved in 3 µL of methanol and mixed with the same amount of matrix (2,5-dihydroxybenzoic acid in 10 mg/ml of methanol, Sigma Aldrich). Each mixture was deposited using the dry droplet method onto a 384 Opti-TOF 123×81 mm MALDI plate (Applied Biosystems) and allowed to dry at room temperature.

**Figure 7:**
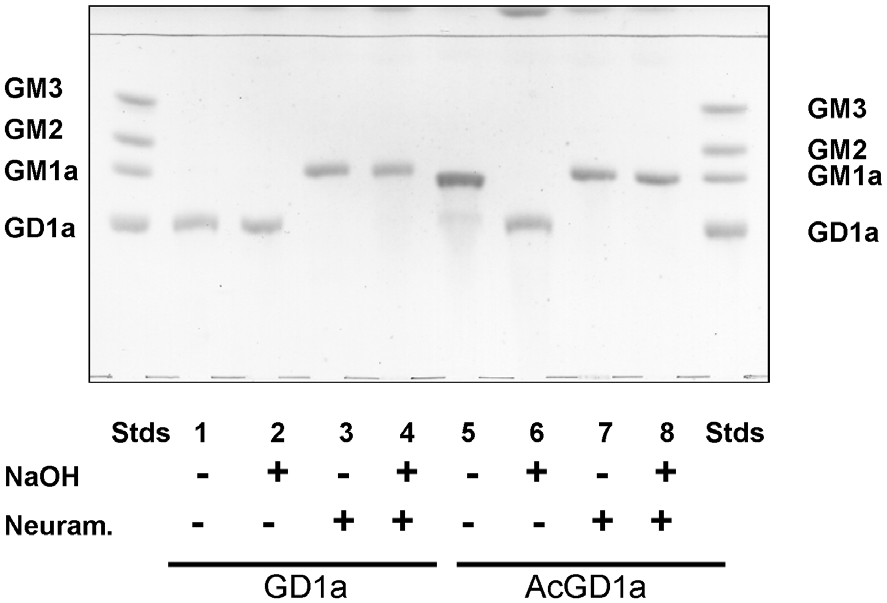
SOAT *O*-acetylates GD1a in the outer alpha-2,3 bound sialic acid. TLC of GD1a and AcGD1a after neuraminidase and alkaline treatment compared to standard gangliosides (GM3, GM2, GM1a and GD1a), Stds. Neuraminidase digestion of AcGD1a (lane 7) or neuraminidase digestion plus alkaline hydrolysis of AcGD1a (lane 8) gives only GM1a as product, demonstrating that AcGD1a is specifically *O*-acetylated in the outermost alpha-2,3 bound sialic acid and not in the inner alpha-2,3 bound sialic acid to the galactose of lactosyl-ceramide.

MALDI-MS was performed using a 4800 Plus MALDI TOF/TOF Analyzer (Applied Biosystems/MDSciex). Spectra were acquired in negative-reflectron mode under an accelerating potential of 20 kV with a Nd:YAG, 355 nm wavelength laser, at 200 Hz frequency. In this analysis, signals between m/z 1000 to 3000 were collected using an external calibration method (4700 Cal Mix, Applied Biosystems).

**Table 1:** Summary of the products obtained in the reactions of different gangliosides with SOAT.

Gangliosides	*O*-acetylated product	Number of *O*-acetylations
GD1a	Yes	1
GD1b	Yes	1
GM1a	nd	nd
GM2	nd	nd
GT1b	Yes	1 or 2

nd = no detected.

### Determination of the *O*-acetylation Sites in the Gangliosides by Electrospray-MS

The gangliosides dissolved in methanol (LCMS grade, Scharlau) were infused into an ion source through an emitter held at a high potential (from −2 to −4.5 kV), to form charged droplets. For electrospray-MS, mass spectra were recorded in negative ion mode on a 4000 QTrap (Applied biosystems/MDSciex) equipped with a Turbo V™ or a NanoSpray^®^II source with ESI probe controlled by Analyst 1.5.1.

Acquisition in EMS scan (500–1200 m/z range) was performed using a declustering potential of −115 V and source gas 1 and curtain gas were set to 10 and 210 respectively. MS/MS data acquisition (Enhanced Product Ion scan) were performed at a scan rate 1000 amu/s, using the same ionization parameters and applying a collision energy of −45 eV with a collision energy spread of 5 eV.

### Determination of *O*-acetylation Sites in AcGD1a by Neuraminidase and Alkaline Digestions

AcGD1a (7 µg) in acetate buffer (50 mM) pH 6, was incubated with *Clostridium perfringens* neuraminidase (1 mU, Sigma-Aldrich) at 37°C, for 1 hour. Neuraminidase digested samples were incubated for one hour at 50°C in methanolic sodium hydroxide (40 µl of 50 mM sodium hydroxide in methanol). Some samples were only treated with neuraminidase or with methanolic sodium hydroxide. In parallel, commercial ganglioside GD1a was treated as reaction control. Samples were desalted with PepClean C-18 spin columns (Thermo Scientific, Rockford, USA). Briefly, the resin from the columns was activated with two consecutive washes with 300 µl of methanol and 300 µl of chloroform/methanol (2/1, v/v). Solvents were removed by spinning down the columns in a bench-top microcentrifuge at 200×*g* for 1 minute. The resin was equilibrated with chloroform/methanol/water (3/48/47, v/v/v). Samples from the digestions were resuspended in chloroform/methanol/water (3/48/47, v/v/v), loaded into the columns and spinned down. The columns were washed 3 times with 300 µl of water. Samples were eluted from the columns with 3 washes with 300 µl of methanol and concentrated on a speed-vac evaporator. A TLC was run as described previously, to monitor digestion patterns in the different treatments and to characterize the *O*-acetylated sialic acids.

### Identification of the *O*-acetylated Hydroxyl Group in the Sialic Acid by NMR Spectroscopy

Samples of ganglioside, either *O*-acetyl-GD1b (∼2 mg) or GD1b (∼1 mg), were deuterium exchanged by repeated drying under vacuum from deuterated methanol. The samples were then dissolved in 550 µl of a mixture of DMSO-d_6_/D_2_O (98∶2) for NMR analysis. High-resolution NMR spectra were registered on a Bruker spectrometer operating at 16.4T (proton Larmor frecuency of 700 MHz) at 295 or 298 K. The residual water signal was suppressed by presaturation of the water resonance at ∼3.48 ppm. For quantitative 1D ^1^H NMR spectra 128 or 256 scans were recorded using an interscan delay of 9 s, a spectral width of 20 ppm, and a 3 s acquisition time. Phase sensitive 2D double quantum filtered correlation spectroscopy COSY [27] and and total correlation spectroscopy (TOCSY, with a DIPSI2 isotropic mixing scheme for 80 and 150 ms) [28] spectra were recorded using 200 indirect increments (complex points), an indirect spectral width of 8 ppm centered on the water resonance, and 64 scans per increment, resulting in total acquisition times of 6.5 h each. 2D sensitivity-enhanced and multiplicity-edited ^1^H-^13^C heteronuclear single quantum coherence (HSQC) spectra [29], [30] were recorded using an indirect (^13^C) spectral width of 140 ppm centered at 70.7 ppm, with 512 indirect increments and 80 scans per increment, resulting in a total acquisition time of 18 h.

All free induction decays (FIDs) were processed with exponential multiplication (0.2 Hz line-broadening) and the ^1^H/^13^C chemical shifts referenced to internal DMSO (2.54/40.45 ppm). Chemical shift (δ) values are reported in parts per million (ppm). Coupling constant values (*J*) are reported in hertz (Hz), and spin multiplicities are indicated by the following symbol: s (singlet), d (doublet), t (triplet), q (quartet), m (multiplet).

## Results and Discussion

### SOAT Synthetizes AcGD1b

Initial reactions with the ganglioside GD1b and the enzyme SOAT in the buffer described previously [23] did not produce any *O*-acetylated compound. Gangliosides are amphipathic compounds that form micelles in aqueous solution [31], which limits the accessibility of gangliosides to the modifying enzymes. In order to increase ganglioside solubility we tried different approaches, using detergents (SDS or Triton X-100), as previously described with other ganglioside modifying enzymes [32]. However, these detergents did not work in our reaction conditions. Saito et al (13) described that the activity of *Arthrobacter ureafaciens* sialidase on ganglioside GM1a increased after adding sodium cholate to the reaction buffer [33]. In 50 mM MES buffer pH 6.5 and 0.05% (w/v) sodium cholate, we obtained a product with a different R_f_ in TLC to commercial GD1b that we characterized as AcGD1b as described below. We determined the concentration of AcGD1b as a percentage (%) of the commercial GD1b added to the reaction. AcGD1b concentration increased in parallel with the sodium cholate (from 0% with no sodium cholate, to 6.5% with 0.05% sodium cholate, and to 11% with 0.2% sodium cholate). Because of the low solubility of sodium cholate at pH 6.5, we tried MES buffer, pH 7 for a possible improvement in the concentration of AcGD1b. After 3 h of reaction at 37°C in MES buffer pH 7, the amount of AcGD1b produced increased in parallel with the increase in sodium cholate concentration and was higher than at pH 6.5 (0% with no sodium cholate, 39% with 0.05% sodium cholate, 75% with 0.1% sodium cholate and 55% with 0.2% sodium cholate. Thus, the highest yield was obtained with a concentration of SOAT of 100 mU in 50 mM MES pH 7 and a sodium cholate concentration of 0.1% (w/v). When the reaction time increased from 3 to 6 h, the yield of AcGD1b reached 90% (900 µg of product/1 mg of GD1b). The yield of the synthesis obtained with SOAT (ES, Figure 1A was much higher than the obtained with the chemical synthesis (CS, Fig. 1A). Besides, the enzymatic method resulted in a single product, compared with the multiple products obtained by CS.

### SOAT *O*-acetylates the Alpha-2,8 Bound Sialic Acid in GD1b to Produce Neurostatin

To determine the number of transferred *O*-acetyl groups in semi-synthetic AcGD1b, we compared MALDI-TOF/MS (negative mode) data obtained for gangliosides GD1b and AcGD1b (spectra not shown). The spectrum of GD1b showed various peaks (m/z) of 1818.1; 1836.1; 1858.0; 1864.1; and 1886.4. The first three peaks (1818.1; 1836.1 and 1858.0) correspond to GD1b that contains a ceramide with a long chain base (LCB) of 18∶1 and fatty acid (F.A.) of 18∶0 in a monoanionic dehydrated form [M-H_2_O-H]^−^, in monoanionic form [M-H]^−^ and in a monoanionic form with a sodium (Na^+^) [M-Na^+^-H]^−^, respectively. The peaks of 1864.1; and 1886.4 correspond to GD1b that contains a ceramide with a LCB of 18∶1 and F.A. of 20∶0 in monoanionic [M-H]^−^ form and in a monoanionic form with a sodium (Na^+^) [M-Na^+^-H]^−^, respectively. The spectrum of AcGD1b showed three major peaks (m/z) at 1900.1, 1906.1, and 1928.1. The first peak corresponds to the monoanionic form of mono-*O*-acetylated GD1b that contains a ceramide with a LCB of 18∶1 and F.A. of 18∶0 with a sodium (Na^+^) [M-Na^+^-H]^−^. The peaks of 1906.1; and 1928.1 correspond to the mono-*O*-acetylated GD1b that contains a ceramide with a LCB of 18∶1 and F.A. of 20∶0 in monoanionic form [M-H]^−^ and in a monoanionic form with a sodium [M-Na^+^-H]^−^ respectively. There was a difference (m/z) of 42 Da between the peaks for gangliosides GD1b and AcGD1b in all species, corresponding to a single *O*-acetylation.

The position of the *O*-acetyl group in AcGD1b was characterized by negative electrospray-MS (Fig. 2B and Fig. S1B), compared to the GD1b spectrum (Fig. 2A and Fig. S1A). The fragmentation spectrum of GD1b showed one peak with 2 negative charges [M/2-H] = 918.08 corresponding to the species (m = 1836.16) that contains a ceramide with a LCB of 18∶1 and F.A. of 18∶0 (Fig. 2A). The fragmentation spectrum of AcGD1b showed one peak with 2 negative charges [M/2-H] = 938.64 (Fig. 2B), corresponding to the mono-*O*-acetylated GD1b species (m = 1877.28), that contains a ceramide with a LCB of 18∶1 and F.A. of 18∶0. The low molecular weight peak (m/z) of B_0_ = 290.16 was present in both spectra (Fig. 2A and 2B) and corresponds to sialic acid. However, the peak of B_1_ = 332.16 (m/z) was only present in the spectrum of AcGD1b and corresponds to the *O*-acetylated sialic acid. The peak of B_2_ = 581.28 present in the spectrum of GD1b that corresponds to a fragment of two bound sialic acids (NeuAcα2-8NeuAc) was absent in the spectra of AcGD1b. Instead of that peak, there is a peak of B_2_ = 623.28 in the spectra of AcGD1b (Fig. 2B) that has an additional 42 Da (a single *O*-acetylation) compared with B_2_ in the spectrum of GD1b, that corresponds to a fragment of two bound sialic acid with a single *O*-acetylation in one of them (*O*-acetyl-NeuAcα2-8NeuAc), demonstrating that one of the sialic acids is mono-*O*-acetylated. The presence of a peak of 1545.84 (in Fig. 2B) corresponds to the ganglioside GM1a containing a ceramide with a LCB of 18∶1 and F.A. of 18∶0, indicating that *O*-acetylation occurred on the outer sialic acid. The same result was obtained comparing the fragmentation spectrum of GD1b (d18∶1/20∶0, Fig. S1A) with the spectrum of AcGD1b (d18∶1/20∶0, Fig. S1B). In conclusion, the enzymatic reaction produced a single product, corresponding to Neurostatin [(Galβ1-3GalNAcβ1-4(*O*-acetyl-NeuAcα2-8NeuAcα2-3)Galβ1-4Glcβ-Cer].

### SOAT *O*-acetylates GD1b in the C9 Position of the Alpha-2,8 Bound Sialic Acid

Once the location of the new *O*-acetyl group at the outer sialic acid unit was established by MS, we used NMR spectroscopy to determine the carbon position bearing this group. The structures of GD1b and AcGD1b are shown in Fig. 3. The NMR experiments were performed using DMSO-d_6_/D_2_O (98∶2) as solvent, because it is known that gangliosides do not aggregate in DMSO, thus allowing high resolution experiments [34].^ 1^H NMR spectra of GD1b and AcGD1b are shown in Fig. 4A. The spectrum of GD1b was in agreement with that reported by Acquotti *et al* [34] in DMSO-d_6_. The chemical shifts and the corresponding assignments for the spectrum of AcGD1b are provided in Table S1 (see Supplemental Data). The resonances were assigned using COSY, TOCSY (Fig. S2) and HSQC (Fig. 4C) experiments, and by comparing the 1D ^1^H NMR spectrum with that of previously characterized starting GD1b [34] and confirmed here after analysis of the 2D NMR experiments recorded. The anomeric and acetyl regions of the ^1^H NMR spectra of GD1b and AcGD1b are compared in Fig. 4B. Both compounds showed a similar pattern for anomeric protons, except for a new doublet integrating for one proton, which appeared at 4.26 ppm in the spectrum of AcGD1b. This signal corresponds to one of the two protons bound to the carbon C-9 of the terminal sialic acid residue (Sial B, Fig. 3), which bears the new acetyl group. The large *J* (10.06 Hz) corresponds to the geminal coupling constant between these two protons (H-9a and H-9b, Fig. 4B, anomeric region) of the Sial B residue. Moreover, the presence of a new singlet at 2.03 ppm (corresponding to a CH_3_ group by the multiplicity-edited HSQC, not shown) confirmed acetylation (Fig. 4B, acetyl region). The multiplicity-edited HSQC experiment also indicated that the proton at 4.26 ppm, together with the other geminal proton signal at 3.85 ppm (Figure S2) that is not resolved in the 1D spectrum, is bound to a primary carbon (Figure 4C). The dramatic change of chemicals shifts of this CH_2_ group (Table S1) is a direct consequence of the acetylation. In conclusion, the enzymatic reaction produced a single product, corresponding to Neurostatin, that is the ganglioside GD1b with a single *O*-acetylation in the C-9 of the outer sialic acid [Galβ1-3GalNAcβ1-4(9-*O*-acetyl-NeuAcα2-8NeuAcα2-3)Galβ1-4Glcβ-Cer].

### SOAT Synthetizes AcGD1a

To detemine whether SOAT induced *O*-acetylation of alpha-2,3 bound sialic acids, we used GD1a ganglioside as substrate. This ganglioside has two alpha-2,3 bound sialic acids, one bound to the outer galactose and the second bound to the galactose close to the ceramide. Using the same reaction conditions as described for GD1b (100 mU of SOAT in 50 mM MES pH 7 with 1 mM GD1a and 0.1% sodium cholate (w/v)) a single band, named AcGD1a, was obtained with different R_f_ in TLC than GD1a (Fig. 5). AcGD1a represented more than the 70% of the GD1a ganglioside used as starting product for the reaction.

### SOAT *O*-acetylates GD1a in the Alpha-2,3 Bound Sialic Acid

MALDI-TOF/MS spectrum in negative mode from GD1a showed 3 major peaks (spectra not shown). The first two peaks (m/z = 1818.0 and 1858.0) correspond to GD1a that contains a ceramide with a LCB of 18∶1 and F.A. of 18∶0 in a monoanionic dehydrated form [M-H_2_O-H]^−^ and in a monoanionic form with a sodium (Na^+^) [M-Na^+^-H]^−^, respectively. The other peak (m/z = 1886.0) correspond to GD1a that contains a ceramide with a LCB of 18∶1 and F.A. of 20∶0 in a monoanionic form [M-H]^−^. MALDI-TOF/MS spectrum from AcGD1a showed 3 major peaks. The first peak (m/z = 1900.1) corresponds to GD1a with a single *O*-acetylation that contains a ceramide with a LCB of 18∶1 and F.A. of 18∶0 in a monoanionic form with a sodium (Na^+^) [M-Na^+^-H]^−^. The other two peaks (m/z = 1906.1 and m/z = 1928.1) correspond to GD1a that contains a ceramide with a LCB of 18∶1 and F.A. of 20∶0 in a monoanionic form [M-H]^−^ and in a monoanionic form with a sodium (Na^+^) [M-Na^+^-H]^−^ respectively. In conclusion, MALDI-TOF/MS spectrum from AcGD1a showed that the compound has a single *O*-acetylation.

The position of the *O*-acetyl in AcGD1a was characterized by negative electrospray-MS (Fig. 6B and Fig. S3B), compared to the GD1a spectrum (Fig. 6A and Fig. S3A). The fragmentation spectrum of GD1a showed one peak with 2 negative charges [M/2-H]^−^ = 917.5 corresponding to the species (m = 1835.0) that contains a ceramide with a LCB of 18∶1 and F.A. of 18∶0 (Fig. 6A). The fragmentation spectrum of AcGD1a showed one peak with 2 negative charges [M/2-H]^−^ = 939.1 (Fig. 6B), corresponding to the mono-*O*-acetylated GD1a species (m = 1878.2), that contains a ceramide with a LCB of 18∶1 and F.A. of 18∶0. The low molecular weight peak (m/z) of B_0_ = 290.1 was present in both spectra (Fig.6A and 6B) and corresponds to sialic acid. However, the peak of B_1_ = 332.2 (m/z) was only present in the spectra of AcGD1a and corresponds to the *O*-acetylated sialic acid, confirming the *O*-acetylation in this saccharide. The presence of two peaks (m/z) of 1544.9 and 1526.8 in the fragmentation spectrum of GD1a (in Fig. 6A) correspond to the ganglioside GM1 containing a ceramide with a LCB of 18∶1 and F.A. of 18∶0 in a monoanionic form [Y_4_] and in a monoanionic dehydrated form [Y_4_-H_2_O] respectively. In the fragmentation spectrum of AcGD1a, apart from the same peaks ([Y_4_-H_2_O] = 1527,9 and [Y_4_] = 1545,9) present in the fragmentation spectrum of GD1a, there are two additional peaks (m/z) of 1587,8 and 1569,8 that correspond to the mono-*O*-acetylated form of GM1 containing a ceramide with a LCB of 18∶1 and F.A. of 18∶0 in monoaionic form [Y_5_] and in a monoanionic dehydrated form [Y_5_-H_2_O] respectively. The same result was obtained comparing the fragmentation spectrum of GD1a (d18∶1/20∶0, Fig. S3A) with the spectrum of AcGD1a (d18∶1/20∶0, Fig. S3B). As the mono-*O*-acetylated product [Y_5_] or [Y_5_-H_2_O] can theoretically be produced by the hydrolysis of either the outermost sialic acid (to give mono-*O*-acetylated GM1a) or by hydrolysis of the inner sialic acid (to give mono-*O*-acetylated GM1b) we could not assign the *O*-acetylated sialic acid by mass spectrometry only.

### SOAT *O*-acetylates GD1a in the Outermost Alpha-2,3 Bound Sialic Acid

We used alkaline hydrolysis with sodium hydroxide and digestions with neuraminidase to determine the position of the *O*-acetylation in AcGD1a (Fig. 7). Alkaline hydrolysis of AcGD1a reduced its R_f_ in TLC to the same R_f_ as the GD1a ganglioside and confirmed that AcGD1a was *O*-acetylated (compare lanes 5 and 6, Fig. 7). Neuraminidase from *Clostridium perfringens* could hydrolyze only the outer sialic acid of ganglioside GD1a and not the sialic acid bound to the galactose close to the ceramide [35], increasing the R_f_ of GD1a to the same R_f_ as GM1a (compare lanes 1 and 3, Fig. 7). Neuraminidase digestion (lane 7, Fig. 7) or neuraminidase digestion plus alkaline hydrolysis (lane 8, Fig. 7) increased the R_f_ value of AcGD1a to the same R_f_ as the GM1a ganglioside, demonstrating that AcGD1a was *O*-acetylated in the outer sialic acid. In conclusion, the enzymatic reaction produced a single product, corresponding to GD1a with a single *O*-acetylation in the outer sialic acid [(*O*-acetyl-NeuAcα2-3Galβ1-3GalNAcβ1-4(NeuAcα2-3)Galβ1-4Glcβ-Cer].

### SOAT Induced *O*-acetylation of the Alpha-2,8 Bound Sialic Acids and the Alpha-2,3 Outermost Sialic Acids of Gangliosides

Table 1 presents a summary of the products obtained by reaction of different gangliosides with SOAT. The enzyme was very specific for *O*-acetylation of the outer alpha-2,8 bound sialic (e.g. in GD1b), and the outermost alpha-2,3 bound sialic acid (e.g. in GD1a). Ganglioside GT1b was *O*-acetylated in the alpha-2,8 bound sialic acid ([NeuAcα2-3Galβ1-3GalNAcβ1-4(*O*-acetyl-NeuAcα2-8NeuAcα2-3)Galβ1-4Glcβ-Cer]) and in the outermost alpha-2,3 bound sialic acid ([*O*-acetyl-NeuAcα2-3Galβ1-3GalNAcβ1-4(NeuAcα2-8NeuAcα2-3)Galβ1-4Glcβ-Cer]). Depending on the reaction time, we obtained more mono*-O*-acetyl GT1b species (short incubation <3 h) or di-*O*-acetylated GT1b ([*O*-acetyl-NeuAcα2-3Galβ1-3GalNAcβ1-4(*O*-acetyl-NeuAcα2-8NeuAcα2-3)Galβ1-4Glcβ-Cer]) (incubation longer than 6 h).

Under the conditions used in this study, SOAT did not induce *O*-acetylation of the sialic acid alpha-2,3 linked to the inner galactose of GM1a and GM2. The resistance of the alpha-2,3-linked sialic acid from the gangliosides GM1a and GM2 to sialidase digestion has been related to the steric hindrance of the sialic acid residue by the substituent on the axial hydroxyl group at position 4 of the galactose residue [36]. Addition of bile salts to the enzymatic reaction mixture reduced the steric impediment and induced the digestion by sialidases of the alpha-2,3 bound sialic acid to the inner galactose in GM1a and GM2 gangliosides [33]. The Galβ1-3GalNAcβ1-4Galβ1-4Glc- or GalNAcβ1-4Galβ1-4Glc- links in these gangliosides hid the sialic acid and prevented SOAT to induce *O*-acetylation. Addition of the bile salt sodium cholate was required for *O*-acetylation by SOAT in the outer alpha-2,3 and alpha-2,8 bound sialic acids of gangliosides. However, addition of sodium cholate did not induce *O*-acetylation of the sialic acid alpha-2,3 linked to the galactose of lactosyl-ceramide, suggesting that the steric hindrance may not be responsible in this case. The reduced accessibility of *O*-acetyltransferases (e.g. SOAT) to this sialic acid and most probably the unstability of the *O*-acetylation could explain that *O*-acetylated GM1a and GM2 gangliosides in the sialic acid have not been found in nature.

This *O*-acetylation method probably could be used for the specific *O-*acetylation of other a, b and c-series gangliosides with alpha-2,8 bound sialic acids and alpha-2,3 bound sialic acids, found in nature [5], e.g. GD2, GT2, GT3, GQ1b and nLD1.

In summary, we have presented a new method to specifically *O*-acetylate ganglioside sialic acids in the C-9 hydroxyl group that permits the large scale preparation of these modified glycosphingolipids. Compared to other protocols described in the literature [12], [13], [14], [15], this method is easy, specific and gives high yields of *O*-acetylated ganglioside.

### Conclusions

We report an easy and specific enzymatic method to obtain Neurostatin and other gangliosides *O*-acetylated in the outer sialic acids. This method permits the large scale preparation of these modified glycosphingolipids.

## Supporting Information

Figure S1 **SOAT** ***O*** **-acetylates GD1b in the alpha-2,8 bound sialic acid.**
(TIF)

Figure S2 **Expansion of the anomeric region of the TOCSY spectrum (with a DIPSI2 mixing for 150 ms) of AcGD1b in DMSO-d_6_/D_2_O 98∶2 at 295 K.**
(TIF)

Figure S3 **SOAT** ***O*** **-acetylates GD1a in the alpha-2,3 bound sialic acid.**
(TIF)

Table S1 **^1^H and ^13^C chemical shifts (ppm) of AcGD1b in DMSO-d_6_/D_2_O 98∶2 at 295K. See** **Figure 3** **in the article for nomenclature.**
(TIF)
