# Supplementary figures and images for: Specific Synthesis of Neurostatin and Gangliosides O-Acetylated in the Outer Sialic Acids Using a Sialate Transferase
Source: PLoS One. 2012 Dec 3;7(12):e49983. doi: 10.1371/journal.pone.0049983 (PMC3513307; doi:10.1371/journal.pone.0049983)

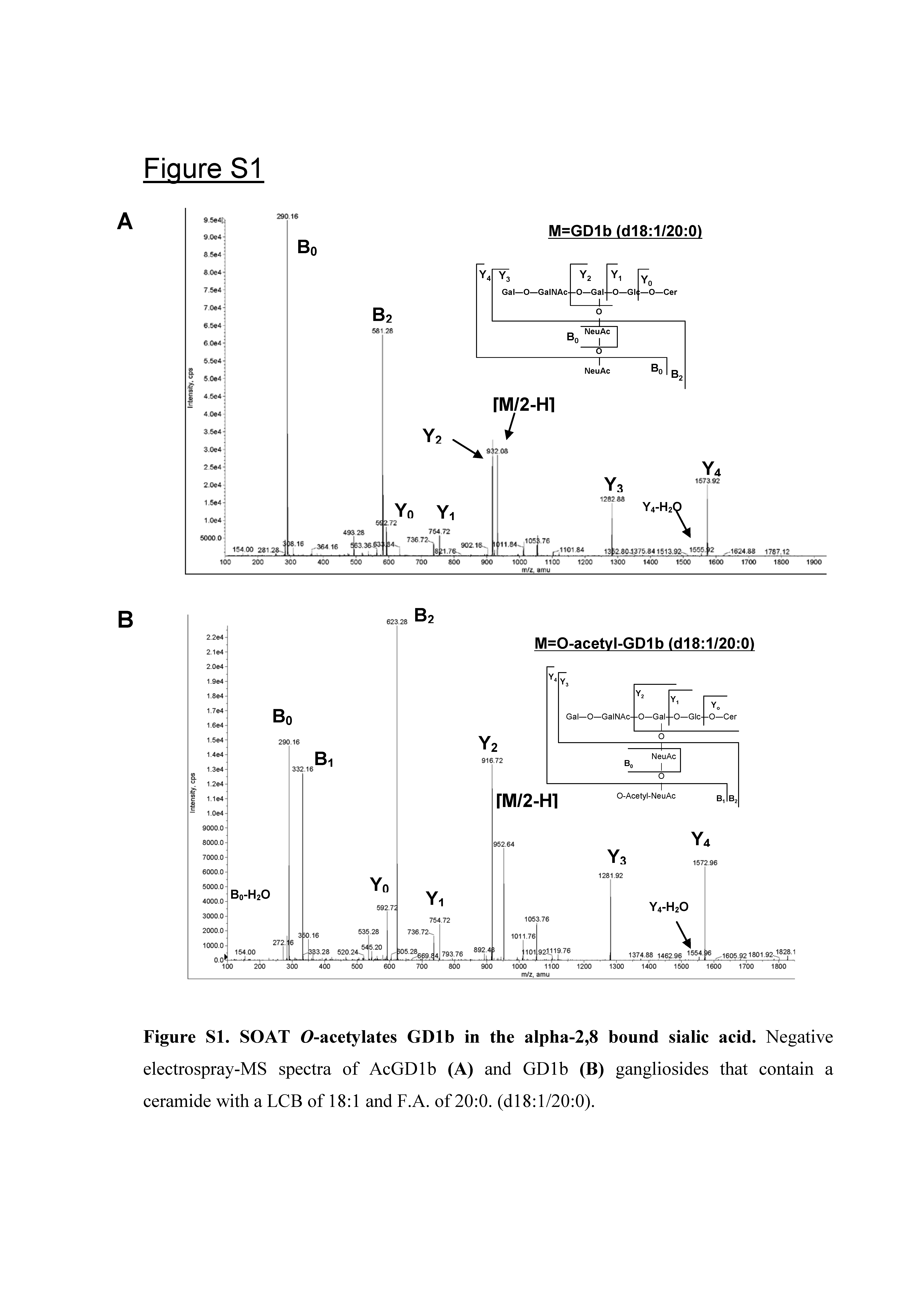

Supplement: Figure S1 — SOAT O -acetylates GD1b in the alpha-2,8 bound sialic acid. (TIF) [file pone.0049983.s001.tif]

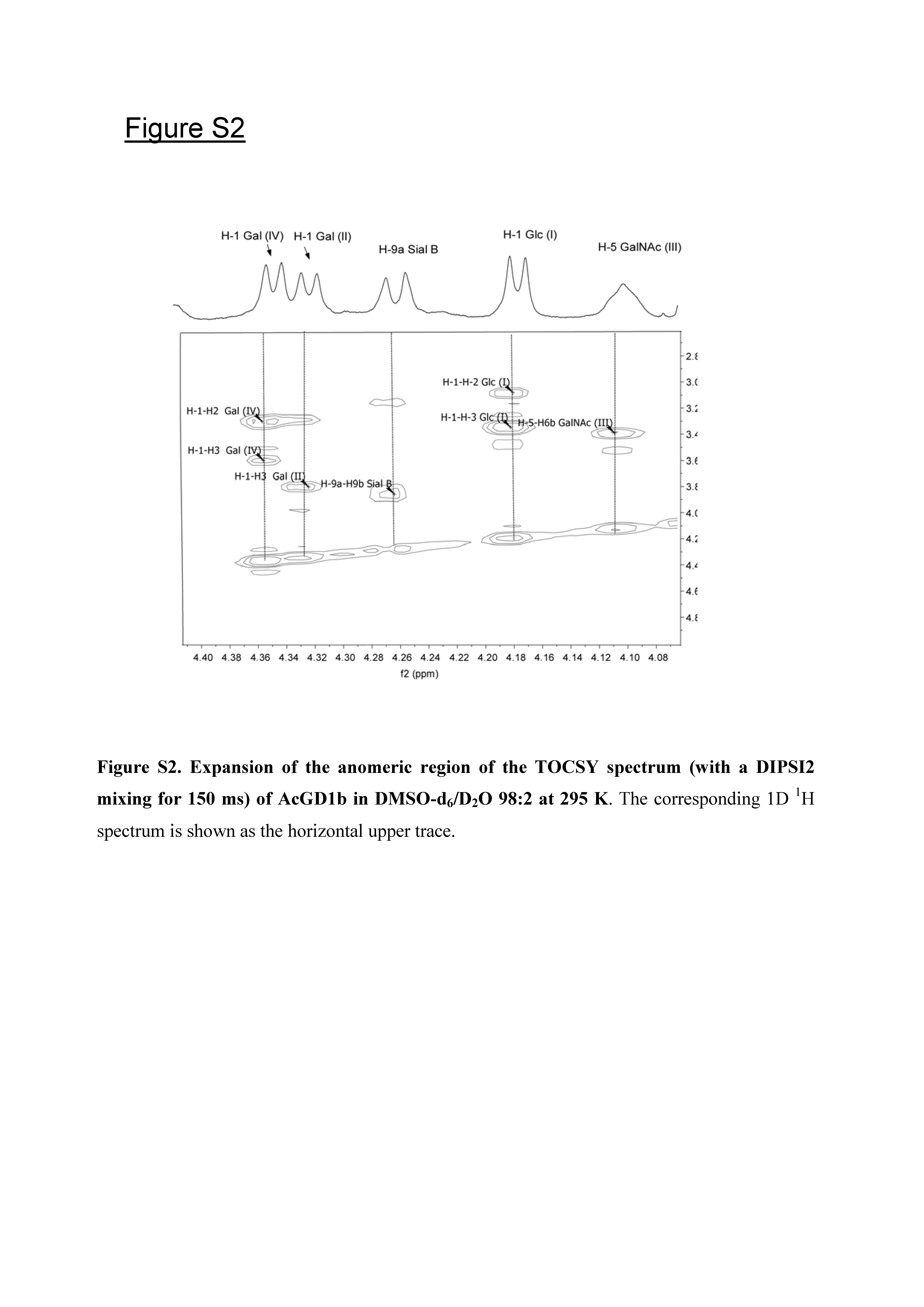

Supplement: Figure S2 — Expansion of the anomeric region of the TOCSY spectrum (with a DIPSI2 mixing for 150 ms) of AcGD1b in DMSO-d6/D2O 98∶2 at 295 K. (TIF) [file pone.0049983.s002.tif]
